# Successful Implementation of Image-Guided Pencil-Beam Scanning Proton Therapy in Medulloblastomas

**DOI:** 10.3390/diagnostics13213378

**Published:** 2023-11-03

**Authors:** Anindita Das, Utpal Gaikwad, Ganapathy Krishnan, Adhithyan Rajendran, Sushama Patil, Preethi Subramaniam, Uday Krishna, Manoj G. Wakde, Srinivas Chilukuri, Rakesh Jalali

**Affiliations:** 1 Neuro-Oncology Cancer Management Team, Department of Radiation Oncology, Apollo Proton Cancer Centre, Chennai 600041, India; dranindita_d@apollohospitals.com (A.D.);; 2 Department of Medical Physics, Apollo Proton Cancer Centre, Chennai 600041, India; 3 Department of Diagnostic and Intervention Radiology, Apollo Proton Cancer Centre, Chennai 600041, India; 4 Department of Pathology, Apollo Proton Cancer Centre, Chennai 600041, India; 5 Paediatric Oncology Cancer Management Team, Department of Radiation Oncology, Apollo Proton Cancer Centre, Chennai 600041, India

**Keywords:** medulloblastoma, intensity-modulated proton therapy, molecular subgroups, craniospinal irradiation, risk stratification in medulloblastoma, pencil-beam scanning

## Abstract

Medulloblastoma is the most common malignant brain tumour in children, while much rarer in adults. Although the prognosis and outcomes have greatly improved in the era of modern multidisciplinary management, long-term treatment-induced toxicities are common. Craniospinal irradiation followed by a boost to the primary and metastatic tumour sites forms the backbone of treatment. Proton therapy has been endorsed over conventional photon-based radiotherapy due to its superior dosimetric advantages and subsequently lower incidence and severity of toxicities. We report here our experience from South-East Asia’s first proton therapy centre of treating 40 patients with medulloblastoma (38 children and adolescents, 2 adults) who received image-guided, intensity-modulated proton therapy with pencil-beam scanning between 2019 and 2023, with a focus on dosimetry, acute toxicities, and early survival outcomes. All patients could complete the planned course of proton therapy, with mostly mild acute toxicities that were manageable on an outpatient basis. Haematological toxicity was not dose-limiting and did not prolong the overall treatment time. Preliminary data on early outcomes including overall survival and disease-free survival are encouraging, although a longer follow-up and data on long-term toxicities are needed.

## 1. Introduction

Medulloblastoma is the most common malignant brain tumour in children [1]. In contrast, adult medulloblastomas are relatively much rarer tumours [2]. The current standard adjuvant treatment for medulloblastoma after maximal safe excision follows a risk-stratified approach, comprising craniospinal irradiation (CSI), with or without concurrent chemotherapy, and a boost to the primary tumour site and any metastatic sites [3,4].

The target volume in CSI includes the whole brain, entire meningeal reflections, complete spinal canal and thecal sac, i.e., the entire neuraxis. Over the last six decades, there has been a major evolution in the planning and implementation of CSI. Despite this, conventional photon-based CSI is associated with a good amount of acute toxicities, since a large volume receives moderate doses of radiation, including a significant amount of vertebral bone marrow irradiation. This in turn leads to the risk of infections and hospital admissions, particularly when combined with concurrent chemotherapy, and ultimately causes undesirable breaks in treatment [5]. Moreover, despite current treatment methods significantly improving the prognosis for medulloblastoma patients, survivors unfortunately often experience several long-term sequelae of treatment.

The physical properties of protons confer well-established dosimetric advantages over any form of modern photon therapy, especially in large, irregular targets like CSI [6]. Due to the scarcity of proton infrastructure and the high cost of proton facilities, generating level 5 evidence is often improbable. Several leading bodies including ASTRO, JASHPP, and a consensus report from the Stockholm paediatric proton therapy conference, therefore, have strongly advocated the use of proton therapy as the treatment of choice in medulloblastomas regardless [7,8]. Modern proton therapy, both due to increasing availability and refinement of planning and delivery techniques, has thus emerged as the preferred alternative whenever available to traditional photon-based radiotherapy for medulloblastoma treatment.

Other treatment paradigms in medulloblastoma also have undergone a lot of scrutiny and changes in the recent past, especially with the increasing integration of molecular profiling into the diagnosis, risk stratification, and subsequent treatment implications. Molecular groups have taken precedence in risk stratifying; on the one hand, the WNT group has been shown to be enough of a good prognostic indicator to warrant de-escalating treatment, while Group 3 and myc-amplification by themselves have been found to be associated with the worst prognosis and hence warrant classifying as high risk regardless of other parameters. The extent of resection being the solitary qualifier for high risk has also come under question and is superseded by molecular groups and other prognostic factors [9]. The benefit of concurrent chemotherapy with intravenous Carboplatin as a radiosensitising agent administered daily before CSI has shown promising results in medulloblastomas with neuraxial metastases (M+ disease), particularly Group 3 high-risk medulloblastomas [10,11]

Our institute, the Apollo Proton Cancer Centre, is India, South-East Asia, and Middle East Asia’s first proton therapy facility. It was inaugurated in January 2019, and we treated our first patient with prostate cancer in Jan 2019, closely followed by our first paediatric patient for craniopharyngioma. We started craniospinal proton therapy also in Jan 2019, first with an adult patient for an intracranial germ cell tumour. Subsequently, our first patient of medulloblastoma was treated with proton CSI in April 2019. Since then, we have treated patients not only from India, South-East Asia, the Middle East, and Africa, but also from Europe, Australia, New Zealand, and South America. We present here our experience of treating consecutive medulloblastoma patients with intensity-modulated proton therapy over a period of 4.5 years since the inception of our treatment facility. Further, we briefly discuss and summarise our findings against those from some of the major studies elucidating the possible benefits of proton therapy in the management of medulloblastoma.

## 2. Materials and Methods

Forty consecutive patients with histologically proven medulloblastoma who received CSI and tumour bed/posterior fossa boost using image-guided, intensity-modulated proton therapy (IMPT) at our centre were included in this study. Each patient’s details were discussed rigorously in an institutional multidisciplinary tumour board before initiating treatment. The study was conducted according to the declaration of Helsinki and the International Conference on Harmonization on Good Medical Practices. Due ethics clearance for this retrospective study was obtained from the institutional ethics committee.

An institutional standard operating procedure was defined and, accordingly, the patients were simulated following a pre-planning audit involving the radiation oncologist (RO), respective physicist, and radiation therapy technologist (RTT). Patients were simulated supine, with arms by the side. A thermoplastic head and shoulder mask (Fiberplast, QFix, Avondale, AZ, USA) with an additional customised neckrest (Moldcare Cushion Qfix, Avondale, AZ, USA) were used for immobilising the brain and cervical spine, and a customised vacloc (full body for children and till mid-thighs for adults) was made for thorax, mid- and lower-body immobilisation. Special caution was taken to avoid air gaps and skin folds at the junction of the immobilisation devices. Non-contrast planning CT with 2 mm slice thickness including vertex to mid-thighs as the region of interest was performed. Contrast-enhanced volumetric sequences of MRI images of the brain and whole spine screening were acquired in the treatment position with a 2 mm slice thickness. For children requiring sedation, the entire process from immobilisation to CT involved an RTT and dedicated paediatric anaesthetist. The same team was maintained for the entire treatment to enhance reproducibility and immobilisation and also to ensure a rapport with the child.

The planning MRI sequences were fused with the planning CT, and the target was drawn on the CT while using the information from the MRI, which was especially beneficial for careful delineation of the inferior extent of the temporal lobes, the cerebellum, cranial nerve exits in the skull base, leptomeningeal deposits (if any), and also to correctly estimate the inferior extent of the spinal cord and thecal sac. Target delineation for CSI followed the standard SIOPE contouring guidelines, with some modifications [12]. For skeletally immature paediatric patients, standard contouring guidelines (SIOP and COG) recommend delineation of the whole brain with dural extensions as the cranial clinical target volume, i.e., CTV brain, and the whole vertebral body as spinal CTV to prevent sharp dose gradients within growing bones that can cause long-term skeletal anomalies. Brain CTV expanded geometrically with a 3 mm radial margin and spinal CTV expanded geometrically with a 5 mm radial margin should be combined to generate the CSI planning target volume, i.e., CSI PTV [12]. Following multiple discussions after the initial few patients, we modified our approach for spinal PTV for this group of patients—the whole spinal canal with nerve extensions, or the “CTV spinal canal”, was expanded by a 5 mm circumferential margin to generate the “PTV spinal canal”, and the whole vertebral body “CTV vertebra” was contoured without any additional PTV margin. The final PTV spine structure was generated by adding the PTV spinal canal and CTV vertebra, and robust planning was carried out while underdosing the anterior mucosal structures. This adaptation allowed for reduced anterior organs at-risk (OAR) doses (oesophagus, midline mucosa, dysphagia/ aspiration at-risk structures, i.e., DARS, etc.) while still avoiding a sharp dose gradient in the vertebral body. For adults and adolescent patients who had attained skeletal maturity, the CTV brain was delineated similarly, covering the whole brain and dural cuffs of cranial nerves, while the CTV spine was delineated covering the entire spinal canal (subarachnoid space) and nerve roots. Special caution was exerted while contouring all the cranial nerve exits and skull base foramina in the CTV brain to ensure adequate coverage of the CSF-containing dural sheaths. The CTV brain expanded by 3 mm and the CTV spine expanded by 5 mm were combined to generate the PTV. For the boost contours, a tumour bed boost was opted for most patients, except for patients with extensive cerebellar leptomeningeal disease in which case a posterior fossa boost was selected. Moreover, other leptomeningeal gross deposits in the brain and/or spine, when present, were also boosted. All OARs were drawn, and standard dose constraints were prescribed. A uniform RBE of 1.1 was accounted for in the treatment planning system, and therefore all prescriptions were given in photon equivalent doses or “GyE”. The usual prescription of CSI ranged from 23.4 to 40 GyE. The primary tumour bed or posterior fossa, as indicated, was boosted to a total dose of 54.8 GyE while large or nodular metastatic lesions in the brain and spine were boosted to 50–54.8 GyE and 45 GyE, respectively.

Robustly optimised IMPT plans were generated for all patients using RayStation (Version 9.0, 10.0, 11.0, RaySearch Laboratories AB, Stockholm, Sweden) treatment planning system (TPS). All pencil-beam scanning proton beam therapy (PBS-PBT) plans were generated using Monte Carlo-based dose optimisation and calculation. Brain plans were generated initially with two posterior oblique beams, and in the later patients with two anterior oblique and one direct posterior beam. Additionally, one to three direct posterior beams for the spine were used depending on the length of the spinal target. Using a robustness margin of 3 mm for translational errors and 3–3.5% for range uncertainties, plans were evaluated for target coverage and OAR exposure. Both contours and radiation plans were approved by the proton peer-review team and patient-specific quality assurance (PSQA) of the finalised plan was undertaken prior to treatment implementation.

Patient position verification was undertaken daily with on-board imaging using a cone beam CT (CBCT) and kilovoltage (KV) X-rays, under the supervision of the treating RO and planning physicist, prior to treatment delivery. Institutional standard operating procedures (SOP) for imaging, image verification, and treatment delivery of CSI had been generated after detailed discussion among RO, physicist and RTT and have been reported previously; these were adhered to for all patients [13], refer supplement. Patients underwent quality assurance CT scans at regular intervals either every 10 fractions or earlier if indicated by CBCT/ clinical/ baseline imaging findings. Every patient was reviewed at least weekly during radiation therapy, and more often if indicated. The first follow-up was scheduled 4 weeks after completion of treatment, and subsequent follow-ups were conducted at 3-monthly intervals for the first 2 years, and less frequently thereafter. Acute adverse events were graded using Common Terminology Criteria for Adverse Events (CTCAE v5.0). Late toxicities, if any, and outcomes including progression and survival were recorded for all patients, with a median follow-up period of 12 months (range 5–34 months).

Statistical analysis was performed using IBM SPSS Statistics 22 for Windows (SPSS, Inc., Chicago, IL, USA). The Kaplan–Meier method was used to extract overall survival (OS), progression-free survival (PFS), and disease-specific survival (DSS) with 95% confidence intervals. (OS, PFS, and DSS were calculated from the date of initial biopsy or surgery as the reference date of diagnosis.) To assess the correlation between the survival parameters and potentially relevant covariates, the multivariate Cox proportional hazard model was fitted to the data.

## 3. Results

### 3.1. Demography and Treatment Details

Forty patients with medulloblastoma received CSI using pencil-beam scanning proton beam therapy (PBS-PBT) at our institute from January 2019 to May 2023. Of these, 38 (95%) were less than 18 years of age. An overview of their demographic data, molecular subgroups, risk grouping, and treatment received is presented in Table 1.

On molecular classification, the majority were Group 4 (20 patients, 50%), followed by SHH (8 patients, 20%), followed by Group 3 and WNT (5 patients, 12.5% each), and 2 patients (5%) remained unclassified. The SHH group was further classified into p53-mutant (2 patients) and p53-wild (6 patients) groups. Risk stratification was undertaken as per the recent molecular-integrated system. Of the 23 patients (57.5%) who were classified as high risk, 18 had metastatic disease in the brain and/or spine and among them, 7 had other additional high-risk factors including age < 3 years (2 patients), molecular Group 3 (4 patients), and SHH p53 mutant (1 patient). Of note, both the infants <3 years of age had metastatic disease at presentation. Of the five patients with WNT medulloblastoma, four (80%) had average-risk disease and only one (20%) had high-risk (M3) disease. In contrast, all five patients with Group 3 medulloblastoma (100%) had high-risk (M3) disease at presentation, often with extensive leptomeningeal metastases. Both patients with p53-mutant SHH medulloblastoma (100%) also had high-risk disease (M3), while two of six patients (33.3%) with p53-wild SHH medulloblastoma had high-risk disease. Twelve of 20 patients (60%) with Group 4 medulloblastoma had high-risk disease, of which five and three patients, respectively, had M3 and M2 disease, while four others had non-metastatic disease but were high risk due to residual disease (three patients) and large cell anaplasia (one patient).

Thirteen (32.5%) of all patients had required general anaesthesia (GA) during simulation, and of these, ten patients (25%) required this throughout the entire course of radiation. Three patients could be treated without GA after a few fractions, once they had become comfortable with the surroundings and personnel.

The median CSI dose prescribed was 35 GyE, and 42.5% received 23.4 GyE, 42.5% received 35 GyE, and 12.5% received 40 GyE (chosen in patients with extensive leptomeningeal disease in brain and/or spine) at 1.67–1.8 GyE per fraction. Twenty-six patients (65%) received concurrent chemotherapy. Fourteen patients did not receive any concurrent chemotherapy. The 18 patients who received weekly Vincristine either alone (14 patients) or in conjunction with daily Carboplatin (4 patients) received a median of five cycles, while a median of 15 doses of chemotherapy was delivered to the 12 patients who received concurrent daily Carboplatin either with daily VCR or as a single agent (8 patients).

### 3.2. Dosimetry of CSI Plans

The CSI contours for all 36 patients who were of age 14 years or younger included the entire vertebral bodies, as laid down in the COG-SIOPE guidelines. In addition, one older male child was 15 years of age but Tanner’s stage 3 as per the endocrinologist’s evaluation, with ongoing skeletal growth, and therefore his CSI contours also included the entire vertebral body. The planning for these patients was conducted with certain novel modifications to reduce the anterior mucosal structure doses, which was described briefly in the methods and is described further later in the discussion. Among the patients who received vertebra-inclusive CSI (VI-CSI), the dosimetric data were again analysed separately for the subgroup of 17 patients who received an average-risk dose, i.e., 23.4 GyE, versus the 20 patients who received a high-risk dose, i.e., 35 GyE or higher. The three patients who received vertebra-sparing CSI (VS-CSI) all received 35 GyE CSI.

The details of the OAR doses are tabulated in Table 2 for the overall study population as well as separately for VS-CSI and VI-CSI patients, the latter further stratified as per the CSI dose prescribed. Coverage exceeding 98% was achieved both for PTV D95 and CTV D98. The average maximum dose (Dmax) received by the lens was 3.2 GyE, which was achieved while maintaining ≥97% dose coverage of the cribriform plate. The average cochlear Dmax was 28.3 GyE and ranged from 88.8% of the prescription dose in VS-CSI to 94.3% in VI-CSI. The average mean dose (Dmean) to the parotids for the entire population was 4.42 GyE. The Dmax to the pharyngeal constrictors, midline mucosa (oral cavity, oropharynx, larynx, and trachea), and oesophagus received in the VS-CSI vs. VI-CSI patients were 26.7% vs. 82–92%, 26.7% vs. 78–92%, and 8.17 vs. 82–88% of the prescription dose, respectively. Dmean of the lungs, kidneys, heart, and bowel bag in the VS-CSI vs. VI-CSI patients were 3.57% vs. 11.6–12.5%, 1.66% vs. 14.4–14.7%, 0.02% vs. 1.71–2.14%, and 0.37% vs. 4.53–4.57%, respectively. Dose to the gonads was none in the case of male patients, while it ranged from 0.02% vs. 0.86–1.97% in the female patients who received VS-CSI vs. VI-CSI, respectively.

### 3.3. Acute Toxicities

All patients tolerated proton therapy well and completed the planned CSI and boost. The predominant acute toxicities are listed in Table 1. Seventeen (42.5%) patients developed Grade 2 dermatitis; all others had Grade 1 dermatitis (57.5%). There were no instances of Grade 3 dermatitis. Grade 1 mucositis, mainly mild odynophagia, was reported in 11 patients and Grade 2 mucositis in 4 patients; all of these responded to conservative management and did not have any break in treatment. Only a single patient developed Grade 3 mucositis during treatment—a 3-year-old girl child receiving whole vertebral body CSI who had previously received extensive chemotherapy as per the baby brain protocol. This child required adaptive planning with contour modification and a brief 3-day hiatus in CSI.

Median weight loss during treatment was 4% of baseline weight. Three patients lost weight more than 10% of their baseline, all of whom were children on concurrent chemotherapy with Inj. Carboplatin. Only one patient developed Grade 2 weight loss—the same child who developed Grade 3 mucositis.

Clinically significant neutropenia (Grade 2 and above) was seen in 16 (40%) patients—of these, 14 patients had Grade 2 neutropenia and the neutrophil counts spontaneously improved; no treatment break was required. Only two patients had Grade 3 neutropenia, including one child with febrile neutropenia who required intravenous antibiotics and growth factor injections—this child was receiving concurrent daily Inj. Carboplatin as well. Grade-2 thrombocytopenia was noted in only two patients; the remaining maintained normal platelet counts during treatment. No symptomatic thrombocytopenia was recorded, and no patient required platelet transfusions. Only one patient had anaemia significant enough to warrant blood transfusion during CSI. None of our patients had any instance of Grade 4 haematological toxicity. Two patients required brief hospital admissions—one patient for supportive management of poor intake due to which concurrent daily Carboplatin was stopped after nine doses, and one patient due to subacute intestinal obstruction.

### 3.4. Follow-Up and Outcomes

All but one patient received adjuvant chemotherapy after completion of proton therapy; this patient had received extensive chemotherapy before initiating CSI and therefore adjuvant chemotherapy was skipped due to poor bone marrow reserve. Of the remaining patients who received adjuvant chemotherapy, 28 (70%) completed chemotherapy—however, two patients had Grade 4 haematological toxicities (febrile neutropenia, sepsis requiring admission, granulocyte colony-stimulating factors, intravenous antibiotics, and inotropic support) and one patient had a sudden unexplained cardiac arrest during the last cycle of chemotherapy.

At a median follow-up of 23.5 months (range 5–54 months), 37 (92.5%) patients were alive—87.5% were alive with no evidence of disease, 2 patients (5%) were alive with progressive disease, and one patient (2.5%) was alive with partial response, respectively. Of the three (7.5%) patients who died, two (5%) had died from progressive disease, while one (2.5%) had no evidence of disease but died from a sudden unexplained cardiac arrest on his last day of adjuvant chemotherapy that he had received elsewhere. Among the four patients who had progressed, three patients had Group 3 medulloblastoma and high-risk M3 disease at baseline and developed further diffuse leptomeningeal progression in the brain and spine while on adjuvant chemotherapy itself. They subsequently also failed second-line salvage/metronomic chemotherapy and two of them passed away from progressive disease, while one is currently on best supportive care and palliation. One patient with baseline average risk, Group 4 medulloblastoma, developed a focal leptomeningeal-based large, solitary lesion in the left temporal lobe three years after completion of treatment, which was surgically addressed, and subsequently salvage chemotherapy with an Etoposide-based regimen is underway. The child has no evidence of disease at present. There was no instance of local progression in the primary tumour bed.

**Figure 1:**
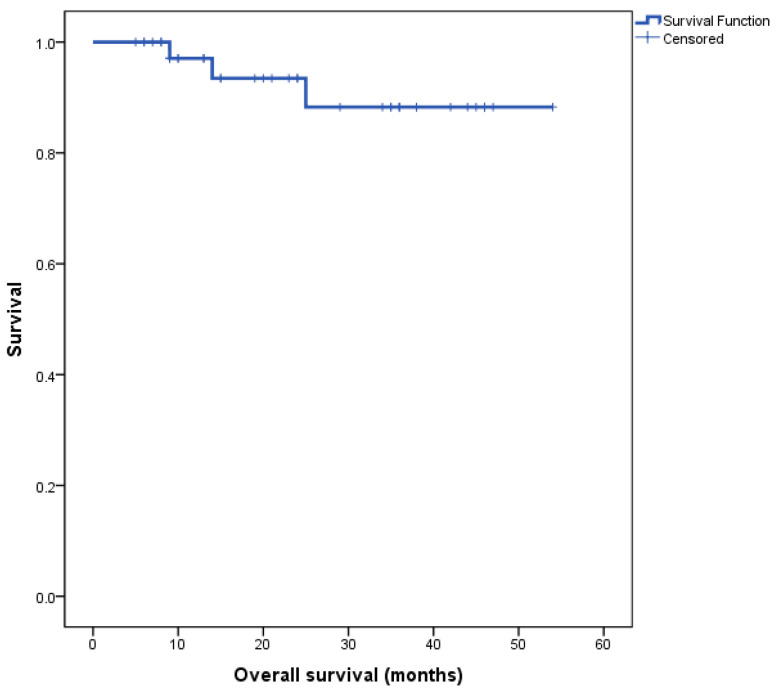
Kaplan–Meier survival curve for all patients, *n* = 40.

The Kaplan–Meier survival curve for the entire cohort’s overall survival (OS) is depicted in Figure 1. Median overall survival (OS) for the entire cohort was not reached at the time of completion of this study.

**Figure 2:**
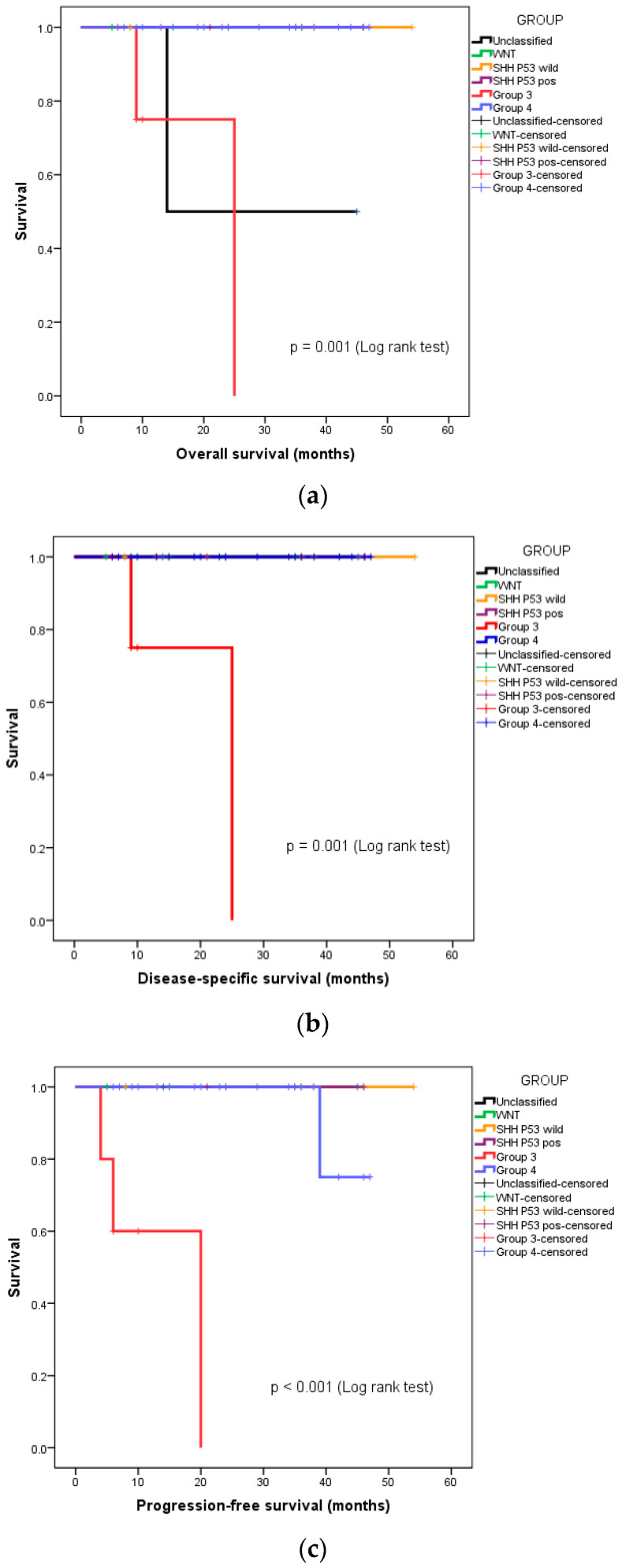
Kaplan–Meier curves depicting survival functions in medulloblastoma patients stratified according to their molecular group: (**a**) Overall survival, (**b**) disease-specific survival, (**c**) progression-free survival.

We also undertook a subgroup analysis of overall survival (OS), disease-specific survival (DSS), and progression-free survival (PFS), based on the molecular group and risk stratification. Both instances of disease-specific mortality were seen in the patients with high-risk, metastatic Group 3 medulloblastoma. The 3-year OS, DSS, and PFS of this group of patients were 88.5%, 91.7%, and 90.8%, respectively. Figure 2a–c depict the separating OS, DSS, and PFS survival curves, respectively, of Group 3 medulloblastoma from the other molecular groups.

**Figure 3:**
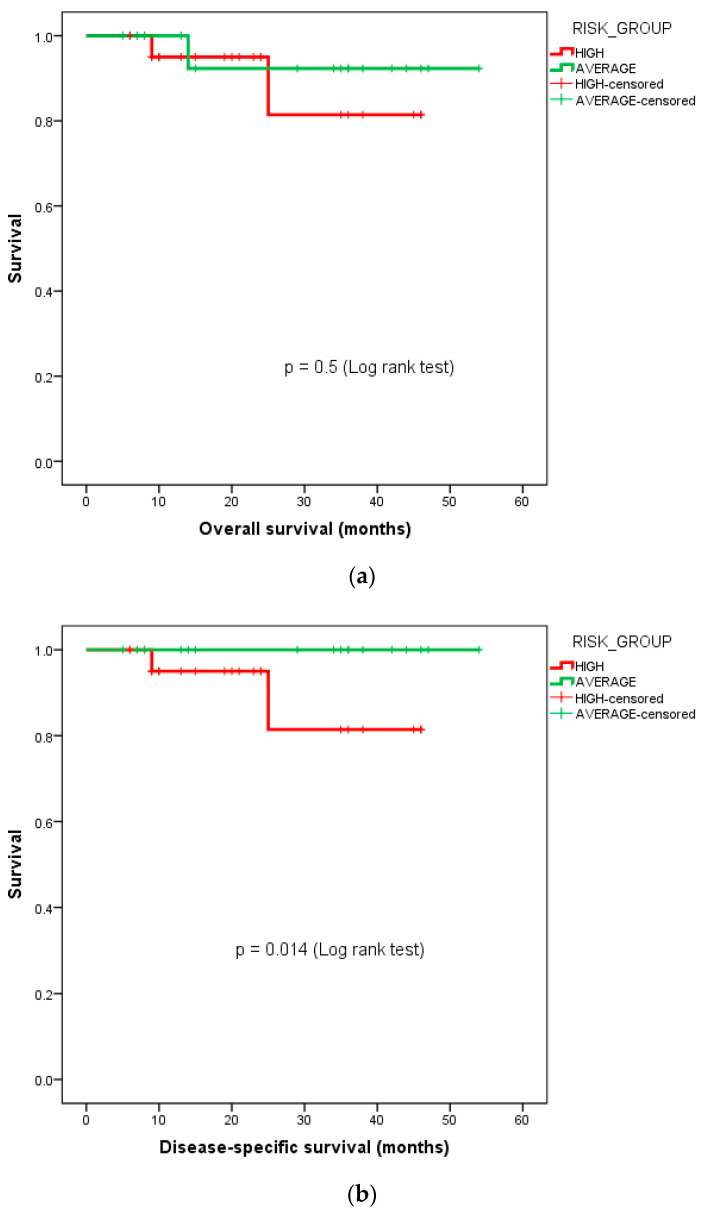
Kaplan–Meier curves depicting survival functions in medulloblastoma patients stratified according to their risk group: (**a**) overall survival, (**b**) disease-specific survival.

The 3-year DSS for the high-risk cohort was 81.4%, while it was 100% for the average-risk cohort. The Kaplan–Meier survival curves for the OS and DSS stratified by the risk groups—i.e., average-risk vs. high-risk subgroups, are depicted in Figure 3.

Ophthalmology, neuroendocrine, neurocognitive, and IQ assessments were routinely carried out at baseline under the respective specialists who are part of our neuro-oncology multidisciplinary cancer management team. Hearing assessment at baseline was not routinely carried out for all patients but was specifically advised for the young children who had received prior chemotherapy before initiation of CSI. We included neuroendocrine reviews as part of the follow-up advice during each clinic-radiological follow-up, and ophthalmological and neurocognitive assessment annually, or earlier if indicated. The long-term outcomes including these parameters and quality-of-life indicators with respect to activities of daily living, education, and employability are not included in the scope of this study and will be separately reported.

## 4. Discussion

An overwhelming majority of paediatric medulloblastomas in the world are diagnosed in low- and middle-income countries (LMICs) [14]. As India and South-East Asia’s first modern proton therapy centre, we have treated more than a thousand patients in the last 4.5 years, among which those suffering from CNS tumours constituted a major proportion. We have previously reported our encouraging preliminary experience in children and young adults, as well as CSI [13,15].

Medulloblastomas are the commonest childhood malignant brain tumours; however, the heterogeneity in biological behaviour and subsequent outcomes has been well demonstrated, and molecular grouping has emerged as the strongest predictor of outcomes [16]. Going further, molecular subgroups have also been demonstrated from genomic profiling and the exact relative prognoses of these are still being studied. The relative distribution of molecular groups WNT, SHH, Group 3, and Group 4 from the literature is known to approximate 10%, 30%, 25%, and 35%, respectively [17]. The relative frequencies were similar in our study, with WNT (12.5%) being the least common, and Group 4 (50%) being the most common type. Thirteen of our paediatric patients required sedation for treatment, of which ten required it daily for the entirety of treatment. The younger age, longer positioning and treatment time, and stringent immobilisation requirements necessitate deep sedation, and therefore the inclusion of a skilled paediatric anaesthetist in the treating team is of paramount importance [18]. With growing familiarity, increased comfort level and rapport with the treatment team, daily sedation for treatment could be avoided in three patients within a week of starting treatment.

CSI forms the backbone of medulloblastoma management. The large, irregular target poses several important challenges in the delivery of radiation, including but not limited to field placements, the management of junctional doses, and minimising integral doses as well as doses to the most proximal OARs like the heart and swallowing structures including the oesophagus, etc., during CSI as well and minimising the doses to intracranial structures during the boost phase. Medulloblastomas, after appropriate management, are associated with good cure rates, and thus questions of long-term survivorship and maintaining the quality-of-life indicators assume more importance, especially since medulloblastoma is predominantly a tumour of the paediatric population. In our patient populace, 95% of the patients were younger than 18 years of age, and all but one patient were younger than 21 years of age. Over the last six decades, the techniques of delivering CSI have evolved considerably even in photon-based RT, and with the increasing availability of proton therapy, it is now not just preferred but also relatively more feasible than even a decade ago. The techniques of proton delivery have also become more conformal, and proton beam therapy with the pencil-beam scanning technique has lent the ability to deliver intensity-modulated proton therapy with improved and more homogenous target coverage as well as superior OAR sparing, compared not only to modern intensity-modulated radiotherapy but also to passive scattering proton beam therapy [19,20]. The mean coverage for our entire cohort as well as the various subgroups exceeded 98% for both PTV D95 and CTV D98, including adequate coverage of the cribriform plate, the underdosing of which is known to cause failures.

**Figure 4:**
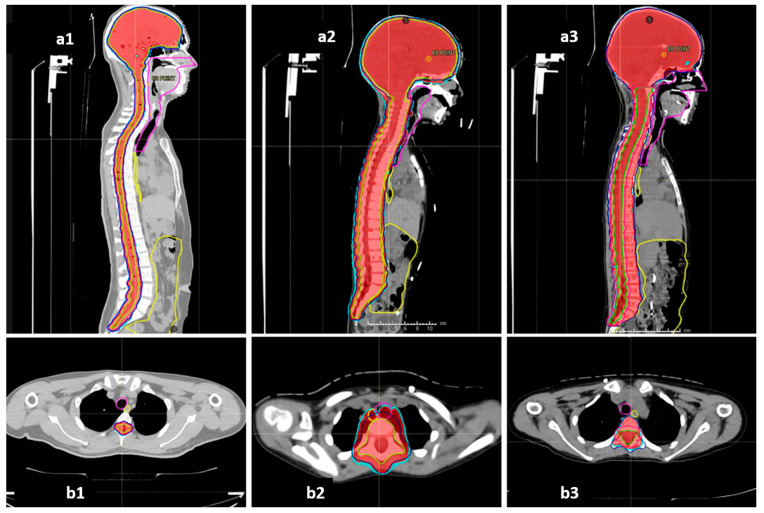
Representative sagittal and axial sections showing the dose colour wash 98% (red) and 95% (cyan) for target coverage and anterior mucosal structures (oesophagus—yellow, midline mucosa—magenta, bowel bag—yellow): (**a1**,**b1**) vertebral-sparing CSI (VS-CSI) plan for skeletally mature patients. The spinal target canal includes the spinal canal and exiting nerve roots with 5 mm margin. Anterior structures are spared well. (**a2**,**b2**) Traditional vertebral-inclusive CSI (VI-CSI) plan for skeletally immature patients (children). The target includes the spinal canal, exiting nerve roots, and entire vertebral body with 5 mm margin. The axial section shows significant spillage of prescription doses into the anterior mucosal structures. (**a3**,**b3**) Modified vertebral-inclusive CSI (VI-CSI) plan for skeletally immature patients (children). The target includes the spinal canal, exiting nerve roots, with 5 mm margin, and the entire vertebral body without any additional PTV margin. The axial section shows adequate and homogenous coverage of the vertebral body between 95–98% isodose, while the anterior mucosal structures are spared well.

The COG-SIOPE guidelines recommend the inclusion of vertebral bodies into the CSI target in skeletally immature children to prevent skeletal abnormalities arising from mismatched growth between the segments of the partially irradiated vertebra. The initial few CSI plans were generated strictly adhering to this; however, after one of them developed severe Grade 3 mucositis, we discussed and noticed that these plans either entailed high dose delivery to the immediately anterior OARs up to 100% of the prescription dose, or sharp, irregular underdosing of the target volume. Thereafter, we adopted a modified novel approach to planning in these pre-pubertal patients to mitigate these concerns. The final spinal target contours concordant with the SIOPE guidelines included the vertebral body (PTV_spine), but the spinal canal with roots with circumferential margin was also separately specified as the target (PTV_canal), which required stricter coverage with the prescription dose. The prescription and target coverage requirements before planning mandated proper coverage of the PTV_canal while routinely prescribing a uniform low dose to the anterior part of the PTV_spine at the vertebral bodies adjacent to the midline mucosal and visceral structures including the pharyngeal constrictors, oesophagus, and heart. The plan was generated keeping these aspects of differential target coverage as part of the optimisation parameters, and during plan evaluation, we verified the vertebral body coverage and independently robustly assessed the PTV_canal coverage accounting for both translational shifts and range uncertainties. This novel approach allowed us to spare the immediately anterior OARs much better while ensuring no or minimal underdosing in the actual target volume at risk as well as no sharp dose gradient within the vertebral body. Representative dose colour wash images of the plans illustrating this further are shown in Figure 4.

The mean dose to the thoracic and abdominal structures anterior to the vertebral body is expected to be lesser in patients where the vertebral body is spared and not included in the CSI target, and the same has been seen replicated by Fukumitsu et al., who demonstrated significantly lower mean doses to thyroid, lungs, heart, oesophagus. and kidneys after vertebral body-sparing CSI versus whole vertebral body-inclusive CSI [20]. In our patients as well, the absolute as well as percentage doses received by the constrictors, midline mucosa, oesophagus, lungs, heart, kidneys, bowel bag, and liver were lower in patients after VS-CSI than in patients with VI-CSI, not only with comparable prescriptions of 35 GyE but also after lower prescriptions of 23.4 GyE in the latter. The recommendation for including the entire vertebral body in the target is based on the rationale and evidence that lateral dose asymmetry within the vertebral body causes scoliosis; however, there are no succinct data to prove that the same is applicable in antero-posterior dose asymmetry as well. In fact, a recent small study used VS-CSI in six children of 3–5 years’ age and found better marrow sparing and retained growth ability, and more importantly did not find any increase in severe spinal anomalies compared to historical data from photon-based CSI where dose uniformity to the vertebra was maintained [21,22]. Since the currently used blanket VI-CSI approach in children effectively undermines quite a few dosimetric advantages of protons as demonstrated here in our study as well as nullifying the possibility of preserving bone marrow and spine growth in a growing child, it warrants further investigation as to the actual necessity of this approach.

The proximity of the eyes to the cribriform plate poses some challenges in ensuring good coverage to the cribriform plate while reducing the lens doses. Initially, we used two posterior oblique fields for the brain following an approach first popularised by Cochran et al., which provided target coverage exceeding 98% while keeping lens doses lower compared to standard bilateral fields [23]. We too have previously published regarding our experience with CSI planning aspects similar to this approach using two non-coplanar posterior oblique beams for the cranial target (gantry: couch 150:330 degrees, 210:30 degrees) and separate direct posterior fields for the spinal target (gantry: couch 180:0 degrees) [24]. As our experience and practices evolved, we adopted a different and distinctive beam geometry which allowed for a simpler setup and improved delivery efficiency while maintaining comparable dose distribution both to the target and OARs, which we have critically appraised and reported [25]. The cranial target exclusively was treated with two anterior oblique coplanar beams (gantry: couch 60:0 and 300:0), while an additional direct posterior beam (180:0) contributed both to the cranial target as well as treated the cervical spine. The middle and lower spine were treated with direct posterior beams as before. The additional beam also allowed redistribution of linear energy transfer of varying proton energies within the cranial target, reducing the radiobiological effectiveness uncertainty in the intracranial OARs. This also gave a leeway to use the posterior oblique beam arrangement in the boost phase, without repeating the original beam arrangement.

An average lens Dmax of 3.2 GyE was noted in our study with this approach and ranged from 1.5 to 6.1 GyE, staying well short of the threshold of 8–20 GyE that has been described to cause clinically apparent opacities, and also considerably less than the reported lens Dmax of 12.4 GyE in the paediatric proton consortium registry for similar optic nerve coverage [23,26].

The cochleae are in close proximity to the CSI target volumes since CSF flow has been noted in studies within the internal auditory meatus close to the cochlea, and as a result, sparing the cochlea at the expense of underdosing the target is not recommended [12,27]. Despite this, the tremendous conformality of proton therapy allows for reducing the cochlear irradiation dose and the consequent dose-dependent sensorineural hearing deficit, both in incidence and severity, and has been shown to translate to cost-effectiveness and a quality-of-life benefit. The cochlear doses during CSI in the average-risk and the high-risk groups were shown to be 46.4 Gy and 50.0 Gy, respectively, with photon-based RT, and reduced significantly with proton therapy to 29.3 Gy and 39.6 Gy, respectively [28]. In our patients, the cochlear doses for children with average-risk and high-risk disease were 22.1 GyE and 33 GyE, respectively, while that for adults was 31.1 GyE.

The dosimetric benefits of proton therapy and the superior OAR sparing do seem to translate to practical benefits; acute toxicities during proton therapy were mostly limited to alopecia, mild dermatitis, and mild mucositis. All our patients had dermatitis, but limited to Grades 1 and 2. The incidence of dermatitis after proton CSI in the available literature has been found to range from 86 to 100% and is not significantly different between protons and photons [29,30,31]. However, there is a definite reported improvement in the incidence and severity of Grade 2 and above mucositis and GI toxicity, with Brown et al. reporting a relative incidence of 26% vs. 71%, *p* = 0.004 between proton CSI and photon CSI [30]. We found a further lower incidence of clinically symptomatic mucositis in our patient cohort where the majority (60%) of our patients had no mucositis or Grade 1 mucositis (27.5%). Only 5% suffered Grade 2 mucositis, and a solitary incidence of Grade 3 mucositis was seen solely in one of the earliest treated children, which was prevented thereafter by the modified approach to VI-CSI planning as described previously and demonstrated in Figure 4(a3,b3) vs. Figure 4(a2,b2). The relative supremacy of IMPT by pencil-beam scanning over passive scattering technology as well as the in-house modifications in target definition and planning as described earlier perhaps explain the further reduced mucositis and GI toxicities in our patients. The haematological toxicities too were only seen to be Grade 3 when in conjunction with an intensive daily Carboplatin chemotherapy regimen. Previous studies also have reported a significant reduction in haematological toxicities after proton CSI rather than photon CSI [32].

Our practice on concurrent chemotherapy also changed with the evolution of the global literature on the same. Initially, until 2021, all our patients received Inj. Vincristine (VCR) weekly during chemotherapy, irrespective of their risk grouping, as per the original Packer’s regimen from COG studies which had been the established standard [3]. In addition to VCR, concurrent daily Carboplatin was selectively added in high-risk patients with Group 3 or extensive leptomeningeal disease, encouraged by the promising outcomes seen with the implementation of the ACNS0332 protocol which showed 19% and 25% 5-year event-free survival benefit in Group 3 medulloblastoma patients with M0 and M+ disease [10,11]. Going forward, with growing evidence of excellent outcomes after adjuvant CSI followed by chemotherapy even with omission of concurrent VCR, as shown by the St. Jude’s group, we eventually stopped the practice of concurrent VCR for patients and only administered concurrent Inj. Carboplatin daily in a select subset of patients with high-risk disease—ones with Group 3, large cell anaplasia, or extensive M2/M3 disease at presentation [4,33]. Accordingly, in the latter half of 2021 onwards, 14 patients who either had average-risk disease or high-risk disease owing to residual lesion only were exempted from concurrent chemotherapy during CSI, and only the subset of high-risk patients as detailed earlier received concurrent daily Carboplatin.

Medulloblastomas are extremely radiosensitive tumours, and although the prognosis and outcome greatly vary with the molecular grouping and metastatic status, the overall long-term outcomes are quite favourable with current multimodality management. Appropriately treated average-risk medulloblastomas have been shown to have a 5-year OS and PFS exceeding 80%, while it sharply drops to <50% in patients who present with metastatic disease at the outset [3]. The 3-year OS, DSS, and PFS of this group of patients were 88.5%, 91.7%, and 90.8%, respectively. Survival and control outcomes have largely been reported as similar between proton and photon CSI. A recent large systematic review by Young et al. comparing proton vs. photon CSI between more than 2000 medulloblastoma patients from 35 studies reported comparable OS (~85–87%), PFS, and patterns of failure for up to a 10-year follow-up period [34]. Infants younger than 3 years have been typically known to fare worse, with a 5-year survival of 30–50% only; however, this is partly also owing to the usual omission, postponing, or de-escalation of CSI due to their age [35,36]. Proton therapy allows for early initiation of CSI and attaining therapeutic doses even in this group. We have treated two infants less than 3 years of age with medulloblastoma, and both received 35 GyE CSI. While one of them is early in the follow-up at 6 months’ post-treatment, the other has been disease-free for 45 months. The worst outcomes in Group 3 medulloblastoma, especially with the presence of metastatic disease at initial diagnosis, were reasserted in our study as well. Of the five children in our cohort with Group 3 medulloblastoma, two had died of progressive disease, two were alive with progressive or residual disease, and one is disease-free at present and on adjuvant chemotherapy. Both the disease-specific deaths in our entire patient group were from this subgroup of patients, again showing the importance of molecular grouping and its impact on prognostication.

We were limited by a relatively short follow-up period. We intend to continue further follow-up on these patients to report further outcomes. The long-term toxicities, including neurocognitive, neuroendocrine, hearing and vision outcomes, fertility, growth, cataracts, to name a few, as well as secondary malignancies, if any, are being followed up during routine reviews and will be separately reported. Superior intellectual outcomes of significant magnitude including stable IQ and better working memory are reported in patients after proton CSI compared to photon CSI and merits strong consideration as a veto factor for preferring protons for CSI, given its long-term implications in employability, functioning, professional and social independence, and ultimately the quality of life, in a group that enjoys excellent long-term survival [34]. Although the hypothalamo-pituitary axis is irradiated as part of the target, and hence the incidence of most neuroendocrinopathies are comparable, a clinically significant reduction in both central and peripheral hypothyroidism is also reported in patients who received proton CSI vs. their photon-treated counterparts, possibly due to a relative resistance of TSH-producing pituitary cells as well as lesser dose to the thyroid gland itself. Moreover, the cumulative incidence of secondary neoplasms at 10 years was also found to be less after proton CSI (2–5% vs. 8%), which again points towards a potentially superior OS in the longer run [34].

The survival outcomes from the literature up to 10 years appear comparable as discussed, but it will be interesting to follow up further to assess differences in OS and all-cause mortality after decades, as a significant increase in cerebrovascular and cardiovascular events is only expected in the third decade post CSI onwards. Given the significant reduction in the doses received by the anterior structures, especially the heart, carotid arteries, aorta, etc., in proton therapy, it is worthwhile to investigate if a branching in the survival curves is noted going forward. With excellent long-term survival as noted regardless of the modality of RT, dedicated comparative health-related quality-of-life studies are the need of the hour and, hopefully, some robust data will be generated globally via the Pediatric Proton/Photon Consortium Registry.

## 5. Conclusions

Modern, image-guided, intensity-modulated proton beam therapy helps in delivering craniospinal irradiation and tumour bed boost to patients with medulloblastoma, with good compliance and tolerance to treatment, while reducing acute toxicities and minimising treatment interruptions. The early outcomes are encouraging for both paediatric and adult patients. There is room for further stratification of treatment, possibly tailored both towards favourable and unfavourable molecular groups. Further conclusive research to prove or refute the necessity of vertebral body inclusion in CSI targets in children can help in further capitalising upon the dosimetric benefits of CSI, at least in select subsets of patients where a vertebral-sparing approach may be adopted.

## Figures and Tables

**Table 1:** Demographics and treatment details.

Demographics and Treatment Details (*n* = 40)	
Median age	7 years (Range 2 to 37 years)
Age distribution	<3 years—2 (5%)
	3–6 years—16 (40%)
	7–14 years—18 (45%)
	15–18 years—2 (5%)
	>18 years—2 (5%)
Sex ratio (Male: Female)	31:9
Molecular group	WNT—5 (12.5%)
	SHH—8 (20%)
	p53 mutant—2 (5%)
	p53 wild—6 (15%)
	Group 3—5 (12.5%)
	Group 4—20 (50%)
	Unclassified—2 (5%)
Risk stratification	Average—17 (42.5%)
	High—23 (57.5%)
	^1^ M+, with/without other high-risk factors—18
	Anaplastic histology—3
	Residual disease > 1.5 cm only—2
CSI dose	23.4 GyE—17 (42.5%)
	35 GyE—17 (42.5%)
	40 GyE—5 (12.5%)
	Others (38.4 GyE)—1 (2.5%)
Concurrent chemotherapy during CSI	Yes—26 (65%)
	No—14 (35%)
Concurrent chemo agents	Weekly ^2^ VCR 1.5 mg/m^2^, capped at 2 mg—14 (53.8%)
	Weekly VCR + Daily ^3^ Carbo 35 mg/m^2^—4 (15.4%)
	Daily Carbo 35 mg/m^2^—8 (30.8%)
Adjuvant chemotherapy	No—1 (2.5%)
	Yes, completed—28 (70%)
	Yes, ongoing—9 (22.5%)
	Yes, stopped due to progression—2 (5%)
Dermatitis	Grade 1—23 (57.5%)
	Grade 2—17 (42.5%)
	Grade 3—0
Mucositis	Grade 0—24 (60%)
	Grade 1—11 (27.5%)
	Grade 2—4 (5%)
	Grade 3—1 (2.5%)
Neutropenia	Grade 0—18 (45%)
	Grade 1—6 (15%)
	Grade 2—14 (35%)
	Grade 3—2 (5%)

^1^ M+: Metastases in brain/spine; ^2^ VCR: Vincristine; ^3^ Carbo: Carboplatin.

**Table 2:** Dosimetric parameters of the proton CSI treatment plans.

	CSI Total	CSI Vertebra Sparing	CSI Vertebra Inclusive		
		All (CSI Dose 35 GyE)	All	CSI Dose 23.4 GyE	CSI Dose ≥35 GyE
***n***	40 (100%)	3 (7.5%)	37 (92.5%)	17	20
**PTV CSI D95 (Avg)**	98.0%	98.4%	97.9%	98.1%	97.9%
**CTV CSI D98 (Avg)**	98.3%	98.7%	98.2%	98.3%	98.2%
**Lens Dmax (Avg)**	3.20 GyE	2.28 GyE (6.51%)	3.27 GyE	3.30 GyE (14.1%)	3.25 GyE (9.29%)
**Cochlea Dmax (Avg)**	28.3 GyE	31.1 GyE (88.8%)	28.0 GyE	22.1 GyE (94.4%)	33.0 GyE (94.3%)
**Parotids Dmean (Avg)**	4.43 GyE	2.13 GyE (6.09%)	4.65 GyE	4.45 GyE (19.0%)	4.78 GyE (13.7%)
**Constrictors Dmax (Avg)**	25.4 GyE	9.41 GyE (26.7%)	26.9 GyE	21.6 GyE (92.3%)	28.8 GyE (82.3%)
**Esophagus Dmax (Avg)**	23.2 GyE	2.86 GyE (8.17%)	25.0 GyE	20.6 GyE (88.0%)	28.8 GyE (82.3%)
**Midline mucosa Dmax (Avg)**	23.2 GyE	9.41 GyE (26.7%)	24.4 GyE	21.6 GyE (92.3%)	27.4 GyE (78.3%)
**Lung Dmean (Avg)**	3.35 GyE	1.25 GyE (3.57%)	3.35 GyE	2.92 GyE (12.5%)	4.05 GyE (11.6%)
**Kidney Dmean (Avg)**	4.02 GyE	0.58 GyE (1.66%)	4.31 GyE	3.38 GyE (14.4%)	5.14 GyE (14.7%)
**Heart Dmean (Avg)**	0.51 GyE	0.01 GyE (0.02%)	0.55 GyE	0.51 GyE (2.14%)	0.60 GyE (1.71%)
**Bowel Bag Dmean (Avg)**	1.25 GyE	0.13 GyE (0.37%)	1.35 GyE	1.06 GyE (4.53%)	1.60 GyE (4.57%)
**Liver Dmean (Avg)**	0.60 GyE	0.09 GyE (0.26%)	0.67 GyE	0.54 GyE (2.31%)	0.79 GyE (2.26%)
**Gonads Dmean (Avg)**					
**Male**	0	0	0	0	0
**Female**	0.40 GyE	0.01 GyE (0.02%)	0.43 GyE	0.20 GyE (0.86%)	0.69 GyE (1.97%)

Avg: average; Midline mucosa: Oral cavity, oropharynx, larynx, and trachea.

## Data Availability

The data presented here are available on request from the corresponding author.
